# Human Papillomavirus and Retinoblastoma: Evidence From a Systematic Review and Meta-analysis of Cross-Sectional Studies

**DOI:** 10.3389/ijph.2023.1605284

**Published:** 2023-07-11

**Authors:** Hong Feng, Yuan Deng

**Affiliations:** ^1^ Department of Ophthalmology, Zhongda Hospital Affiliated to Southeast University, Nanjing, China; ^2^ Department of Ophthalmology, Shanghai Ninth People’s Hospital, Shanghai Jiao Tong University School of Medicine, Shanghai, China

**Keywords:** meta-analysis, cancer, human papilloma virus, HPV, retinoblastoma

## Abstract

**Objectives:** To study the prevalence and the association of HPV infection in retinoblastoma and to determine the most common genotype presented in RB.

**Methods:** Following the PRIMSA guideline, 14 studies reporting HPV infection in RB acquired from six databases were included.

**Results:** The prevalence of HPV from 941 RB samples was 15.6% [95% confidence interval (CI): 7.3–30]. Mexico followed by India and Brazil had the highest HPV prevalence in RB samples, 61.7% (95% CI: 17–93), 22.5% (95% CI: 9–47), and 12.1% (95% CI: 2–52), in order. HPV 16 was the most common genotype presented in RB samples 23% (95% CI: 9–47), followed by HPV 18 10% (95% CI: 3–30) and the combined HPV 16–18 6% (95% CI: 0–50). We did not find a significant association between HPV and RB [odds ratio (OR): 12.2; 95% CI: 0.65–232; *p* = 0.09]. However, after removing the largest-weighted study, a significant association between HPV and RB was observed (OR: 45.9; 95% CI; 8.6–245; *p* < 0.001).

**Conclusion:** HPV prevalence in RB samples was 15% and HPV 16 was the most presented genotype in RB samples. There may be an association between HPV and RB that is needed to be confirmed by high quality future studies. Preventive and treatment measures against HPV infection are essential for the prevention of any possible consequences, in particular, RB.

## Introduction

Among all ocular tumors, retinoblastoma (RB) is known to be the major cause of intraocular tumor presented in the childhood period. The incidence of RB varies from one country to another and is estimated to affect 1 patient/20,000 individuals [1]. Single or multiple presentations of the symptoms and signs such as leukocoria, squint, decreased visual acuity, painful red eye, and orbital cellulitis in children, are considered to be the backbone for RB diagnosis in the early stage of the disease where certain investigations such as computed tomography or biopsy may be contraindicated for avoiding serious complications such as metastasis or radiation-induced second malignancy in high-risk patients [2]. In a large meta-analysis of 314 papers, an improving survival rate of RB was noticed among all countries; however, the survival rate was low in low income countries, which potentially stems from the delayed diagnosis of RB cases in it is early stage [3, 4]. Therefore, appropriate preventive and diagnostic measures should be advocated for to raise the survival rate of RB patients.

In recent years, Human papillomavirus (HPV) has become an important driver of the non-inheritable proportion of many cancers [5]. Such high potentiality for developing malignancies showcases its high ability to alternate the normal cell cycle together with the induced genetic mutations [6]. However, many studies have attempted to study the role of HPV in developing RB [7–9]. In a population-based study of Anand et al. [7] HPV was found in a quarter of RB samples that were obtained from Indian patients. Furthermore, the cross-sectional study of Mohan et al indicated that approximately half of RB tissues contained HPV, while HPV was absent in all the control samples [10]. However, the study of Anotoneli et al. [8] indicated that the prevalence of HPV was higher in the control sample than in the RB samples, 9% and 5%, respectively. Moreover, many studies demonstrated that no evidence of HPV was revealed in RB tissues [11, 12]. Due to the reported heterogeneity in the prevalence of HPV infection in RB and the presence or the absence of association of HPV and RB in many studies, we conducted this meta-analysis which will highlight the possible role of HPV in inducing RB.

## Methods

### Search Strategy

**FIGURE 1:**
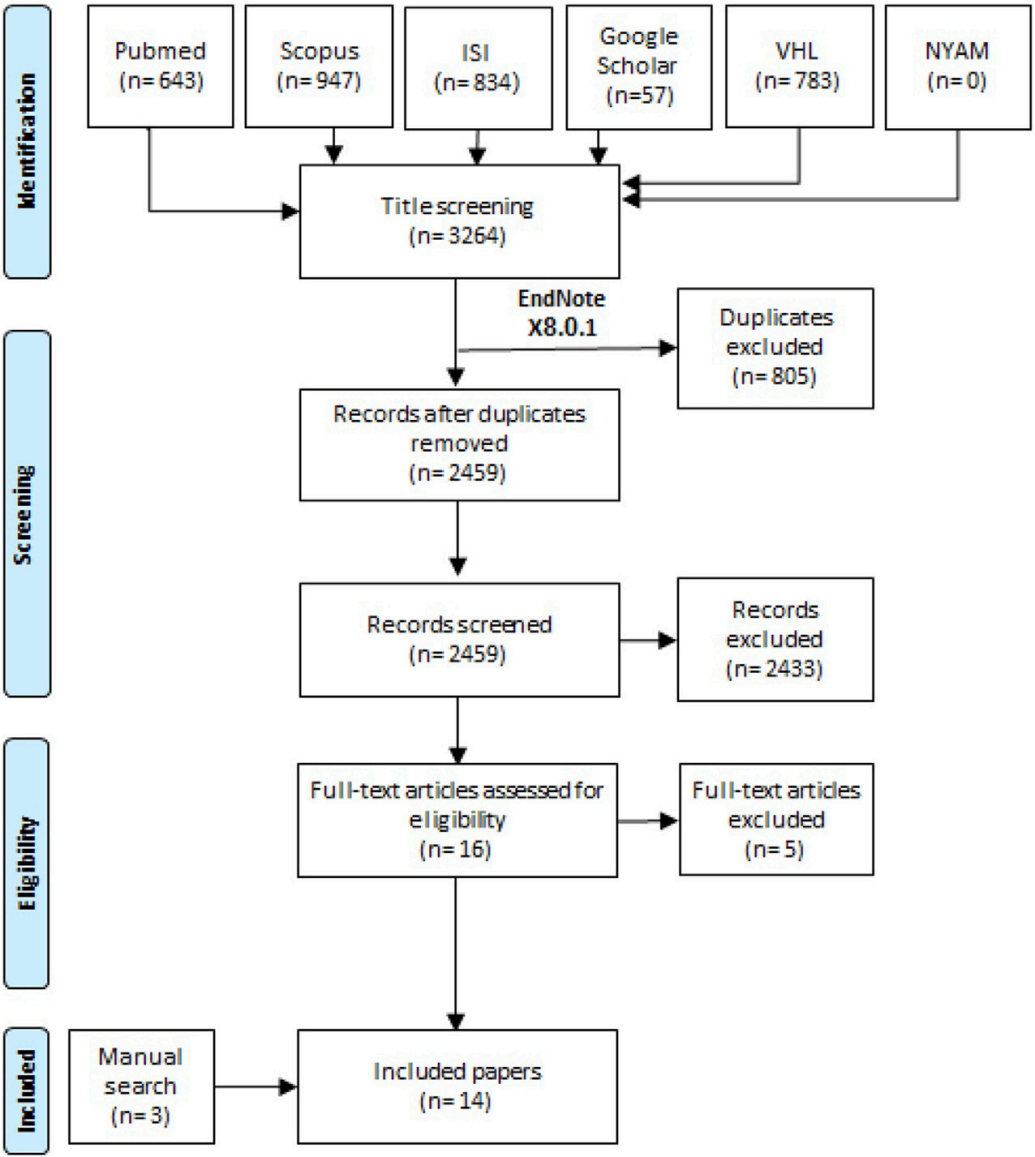
The flow diagram of the study process (Shanghai, China. 2022).

Our systematic review and meta-analysis was developed by following the guidelines of Liberati et al. [13] which are known as the PRISMA checklist. A random search was performed for retrieving the best suitable included studies for performing a search term. After the consensus was finished between the team members for selecting the best keywords; in 27 February, 2022, a search term “(retinoblastoma) AND (“human papilloma virus” OR “HPV”)” was used in all the six databases that were represented in the PRISMA flow diagram (Figure 1).

Inclusion criteria: All papers that reported HPV infection in RB samples were included, whether reported alone or compared to a control population. We made no restrictions against the event of HPV (studies with 0 events were included), language of studies, and the publication year.

Exclusion criteria: We excluded conference papers, previous systematic reviews, books, and duplicate papers including the same patients, which were checked by reviewing the country, recruitment year, and the hospital of the included patients along with the similarity of the authors of the suspected duplicated papers.

After the search term development, one author retrieved all records from the six databases including title, abstract, link, DOI, publication year, and author list of each individual study. The process was performed by Endnote software, and then all records were transported to an Excel sheet for the screening process. Each record was screened from the excel sheet by both authors and a third reviewer when disagreement was presented. Then another step of full text screening was done by both authors for including the best matched studies according to our eligibility criteria. Two experienced members conducted a manual search to retrieve the missed relevant papers by searching the references of any previously published systematic reviews and a hand search in Google Scholar database.

### Data Extraction and Risk of Bias

All the necessary data for the characteristics of the included studies and our outcomes were extracted through an Excel sheet. Our outcomes were: the prevalence of HPV in RB, the common genotype of HPV in RB, and the association between HPV and RB. The characteristics of the included studies were: study ID, male prevalence, country of patients, study design, control definition, age, and the diagnostic method of HPV. Two authors extracted the data and reviewed it for any potential errors. The online tool of the cross-sectional and the cohort studies of the National Institute of Health were retrieved and all studies were assessed for quality into good, fair, or poor domains [14].

### Statistical Analysis

The software used for the analysis was comprehensive meta-analysis software (CMA 3.0). Two effect sizes were pooled, the event rate to analyze the prevalence of HPV in RB, and the odds ratio (OR) to analyze the association between HPV in RB compared to controls along with their 95% confidence interval (CI). Due to the significant heterogeneity we found, we used random effect model in all the analyzed outcomes [15]. Moreover, two tailed *p* values that resulted from Egger’s test were used to report the publication bias in outcomes that had ten or more included studies [16, 17].

## Results

### Search Results and Characteristics of the Included Studies

From the six databases, 3,264 records were retrieved and only 2,459 records were screened using title and abstract screening after excluding duplicate records. We screened 16 full texts for potential eligibility in our study. Finally, we included 14 studies that included three studies from manual search methods (Figure 1) [7–12, 18–25].

There were 14 cross-sectional studies (Table 1). All studies used polymerase chain reaction (PCR) for the identification of HPV except one study, which used the *in situ* hybridization in formalin-fixed paraffin-embedded RB tissues. All studies were of fair criterion (Supplementary Table S1).

**TABLE 1:** Characteristics of the included studies (Shanghai, China. 2022).

Study ID	Study design	Sample size RB	Sample size control	Definition of control	Age (mean (SD))	RB diagnosis	Male prevalence in RB
[7]-India	Cross-sectional	64	—	—	2.5 years	PCR	NR
[8]-Brazil	Cross-sectional	154	44	Normal retina from each embedded tissue block	22.4 months	PCR	74
[9]-India	Cross-sectional	72	—	—	4 years	PCR	46
[18]-India	Cross-sectional	106	—	—	26.7	PCR	55
[10]-India	Cross-sectional	44	30	Non-neoplastic donor retinas	2^a^	PCR	27
[19]-Mexico	Cross-sectional	51	6	Non-RB occular tissue	2 months-8 years^b^	PCR	NR
[20]-India	Cross-sectional	39	42	Donor eyes obtained from the eye bank	41.8 (26.2)	PCR	20
[21]-Mexico	Cross-sectional	39	—	—	<5 years	PCR	NR
[22]-Brazil	Cross-sectional	43	—	—	28.8 months (17)	PCR	21
[23]-India	Cross-sectional	76	—	—	36 months^a^	PCR	NR
[11]-Korea	Cross-sectional	54	—	—	22 months	*In situ* hybridization in formalin-fixed paraffin-embedded retinoblastoma tissues	NR
[24]-Thailand	Cross-sectional	111	12	Coats disease, endophthalmitis, and congenital glaucoma	NR	PCR	56
[26]-Iran	Cross-sectional	61	v	—	28.6 years (17.3)	PCR	32
[12]-Multicenter in North America	Cross-sectional	40	—	—	NR	PCR	23

NR, not reported; RB, retinoblastoma; PRC, polymerase chain reaction.

^a^ Range.

^b^ Range.

### Prevalence of HPV in RB

**FIGURE 2:**
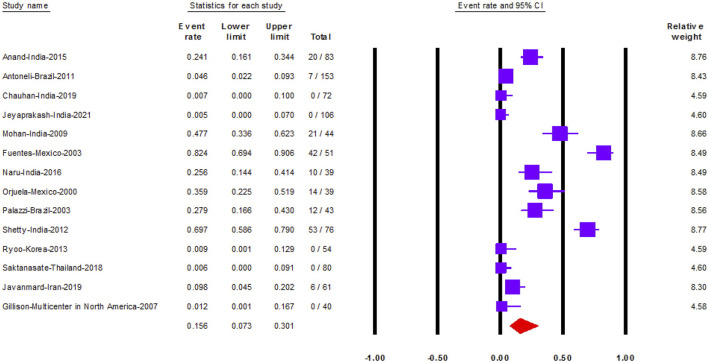
The prevalence of Human papilloma virus in retinoblastoma (Shanghai, China. 2022). Notes: The data is represented with the event rate and the corresponding 95% confidence interval.

Fourteen studies reported HPV prevalence in 941 RB samples. The prevalence of HPV was 15.6% (95% CI: 7.3–30) (Figure 2).

We performed subgroup analysis according to the country of each study. Mexico followed by India and Brazil had the highest prevalence of HPV in RB samples, 61.7% (95% CI: 17–93), 22.5% (95% CI: 9–47) and 12.1% (95% CI: 2–52), in order (Supplementary Figure S1). Moreover, studies conducted in Korea, Thailand, and the multicenter study conducted in North America, indicated that HPV was absent in all RB samples.

**FIGURE 3:**
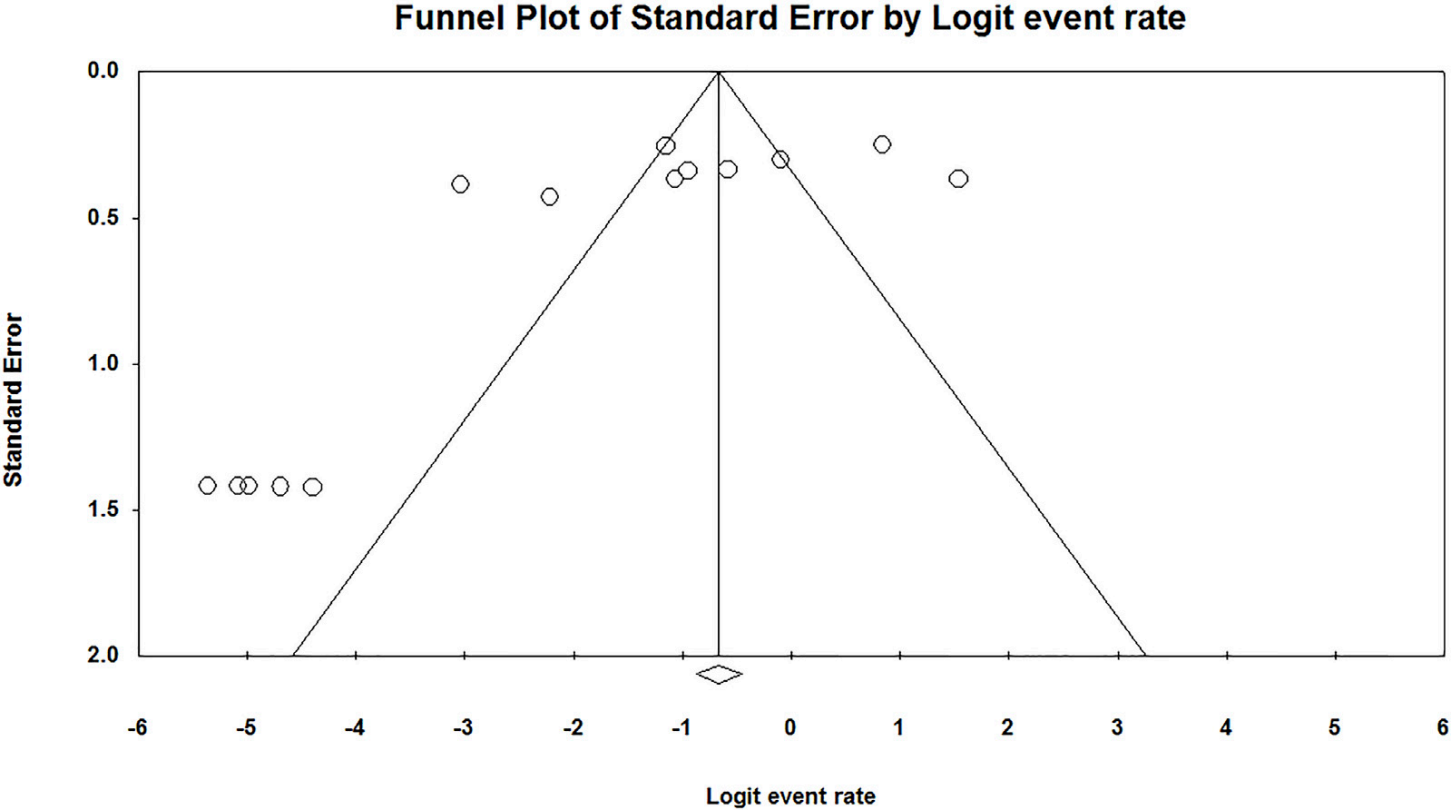
Publication bias of the prevalence of Human papilloma virus in retinoblastoma (Shanghai, China. 2022).

We found a significant publication bias in the prevalence of HPV in RB (*p* = 0.02) (Figure 3).

### Association Between HPV and RB

Five studies compared HPV prevalence in RB samples to controls [8, 10, 19, 20, 24]. However, in our analysis we included only four studies [8, 10, 19, 20] as the fifth study demonstrated no events in the RB group and the control group [24]. Therefore, the study effect size will not play a significant role in changing the results of the other pooled studies according to the Cochrane Handbook of systematic review [26].

**FIGURE 4:**
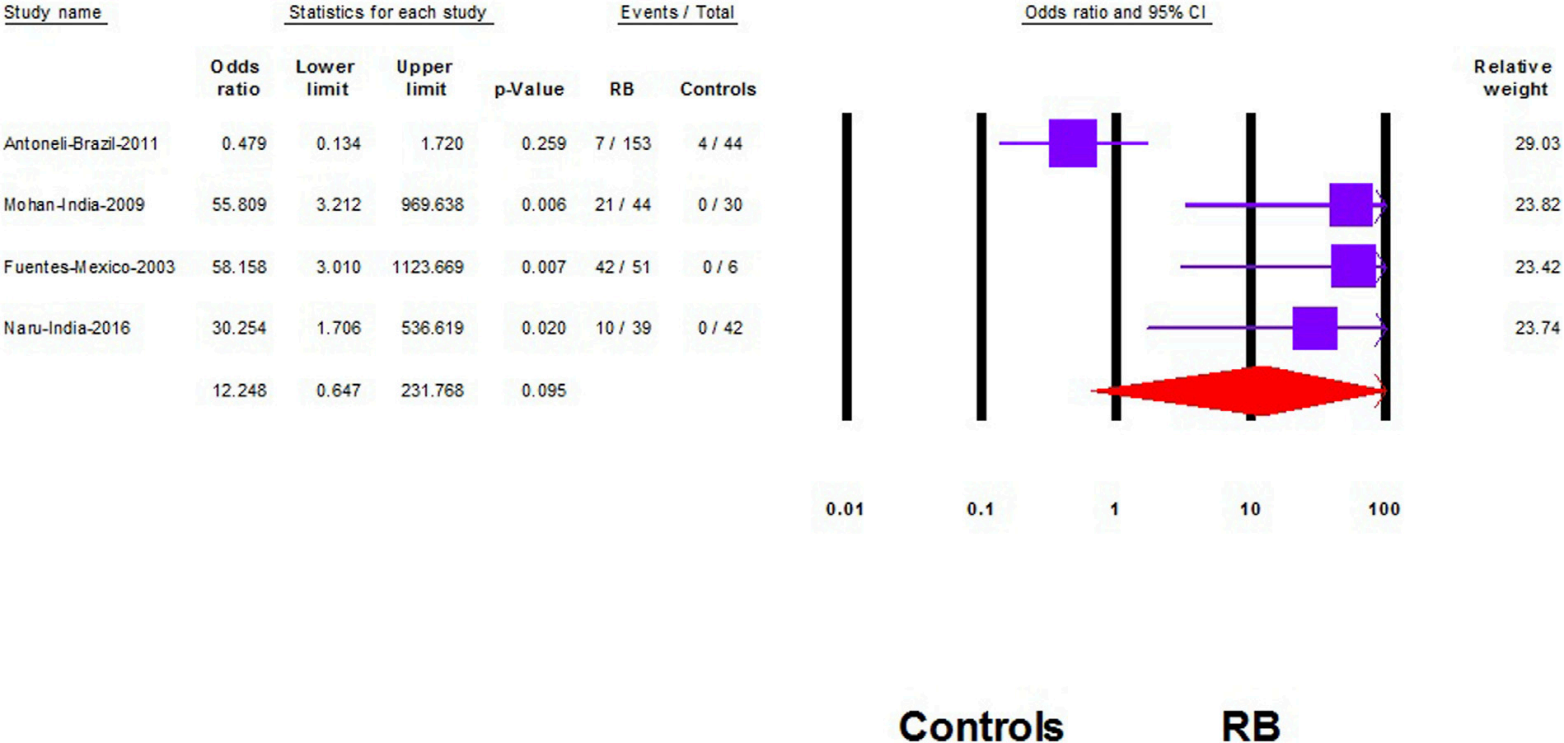
The association of Human papilloma virus and retinoblastoma (Shanghai, China. 2022). Notes: The data are represented with the odds ratio and the corresponding 95% confidence interval.

**FIGURE 5:**
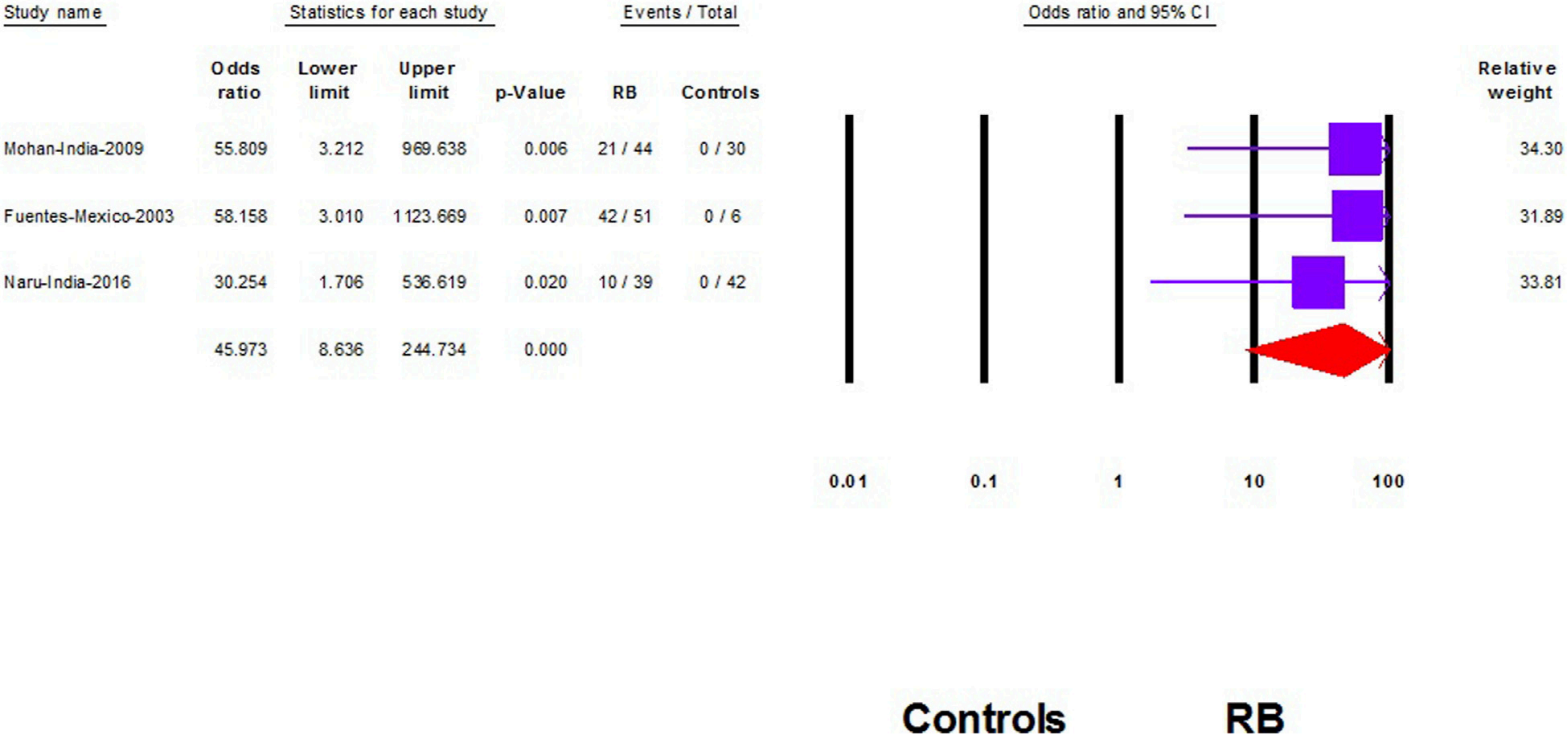
Sensitivity analysis of the association of Human papilloma virus and retinoblastoma (Shanghai, China. 2022). Notes: Association is represented with the odds ratio and the corresponding 95% confidence interval.

We did not find a significant association between HPV and RB (OR: 12.2; 95% CI: 0.65–232; *p* = 0.09) (Figure 4). However, after removing the largest-weighted study, a significant association between HPV and RB was observed (OR: 45.9; 95% CI; 8.6–245; *p* < 0.001) (Figure 5).

### HPV Genotype in RB

HPV 16 was the most common genotype presented in RB samples 23% (95% CI: 9–47), followed by HPV 18 10% (95% CI: 3–30) and the combined HPV 16–18 6% (95% CI: 0–50) (Supplementary Figures S2–S6).

## Discussion

We found that the prevalence of HPV in RB was 15.6% and was the highest in Mexico followed by India. Among all strains of HPV, HPV 16 had the highest prevalence in RB. We did not find an association between HPV and RB; however, the association was prevalent after performing a sensitivity analysis.

Various studies had explored the prevalence of HPV in RB samples and a huge variability in the prevalence among all studies was found. Fuentes et al. [19] indicated that the prevalence of HPV in RB samples from Mexican patients was 82%. Furthermore, the study of Shetty et al, conducted in India, revealed that the prevalence of HPV in RB was 69% [23]. Similarly, Mohan et al. [10] demonstrated that 48% of the Indian samples of RB had HPV infection. Despite this high rate from many studies, the multicenter study that recruited RB samples from North America demonstrated no case of HPV infection in RB samples [12], and no events were found in the Korean RB samples [11]. To our knowledge, low socioeconomic status including education, residence, poor nutritional status, and poverty, together with life style behaviors, plays a vital role in the susceptibility to infection in low and low middle income countries [27, 28]. Of note, Naru et al. [20] demonstrated that RB patients with evident HPV infection were more likely to be of low socioeconomic status and of rural residence than their peers without HPV infection. Moreover, breast feeding duration and mean birth weight were lower in the HPV positive group rather than HPV negative group in Mexican children with RB [21]. These may be explanations as to why no cases of HPV infection were reported in RB samples in high income countries. However, this mechanism does not fully explain the absence of HPV infection in many studies conducted in low middle income countries [9, 18, 24].

In our analysis, HPV 16 was the most common genotype presented in RB tissues. The results were consistent with many studies reported in literature [7, 20, 22, 25]. However, in the study of Orjuela et al. [21] HPV 18 was the most represented genotype in tumor tissue. Moreover, low risk HPV stains were the least reported strains in RB tissue reported from Anand et al. [7]. High risk HPV strains are associated with the development of many cancers [29, 30]. In addition, the high risk HPV stains were associated with RB differentiation and progression [23].

We did not find a significant association between HPV and RB; however the association was presented when we excluded the study of Antoneli et al. [8]. In addition to being the largest weighted study in our analysis which contributed to approximately one-third of the total weight of our pooled OR, the study was different in defining their control status rather than other studies. Antoneli and colleagues selected normal retinal tissue samples from already diagnosed RB cases, not from other normal patients or patients having other diseases than RB such as the other three included studies in the association analysis [10, 19, 20]. Familial history of RB is a significant risk factor for RB development during the childhood period [1]. In RB cases where familial predisposition could not be obtained, searching for the possible cause is a matter of concern among ophthalmologists, especially with the high expected mortality in low and low middle income countries [1]. In the study of Orjuela et al. [21] nearly one-third of RB samples of children without a family history of RB had HPV infection. Shetty and colleagues represented that HPV was prevalent in non-familial cases of RB which supports the direct link of HPV infection and the development of RB [23]. Tumor suppressor genes are essential for preventing cancer development. HPV infection may induce RB through the direct inhibition of the tumor suppressor proteins and the induction of mutation that is necessary for tumor growth and progression [5, 31].

Intensive efforts have been devoted to exploring the possible method of HPV infection in the eye before inducing RB. Different routes of transmission were identified including transmission during pregnancy, sexual contact, autoinoculation transmission, and transmission through fomites [32–34]. Moreover, the mode of delivery is considered a major source of infection at which the neonates acquire infection through an infected birth canal [34]. This is supported by Naru et al. [20] results at which all RB samples infected with HPV were delivered vaginally compared to 72% of the HPV negative cases.

To our knowledge, HPV plays an important role in the deactivation of tumor suppressor genes, in particular pRB and p53 through the E7 and E6 proteins [35]. However, the study of Jeyaprakash et al. [18] indicated that all the DNA extracted from tumor tissues was not positive for HPV infection. Such results demonstrate that further analysis is required regarding the exact mechanism by which HPV induces RB, or the association, are due to other factors such as the genetic susceptibility, socioeconomic status, or family history of RB patients.

Our study had many limitations therefore our results should be taken with caution when interpreted by future studies. Firstly, all studies were cross-sectional in nature, which cannot determine the exact relationship between HPV and RB, therefore more prospective studies with long follow up periods are needed to confirm this association. Secondly, we found a significant heterogeneity between the included studies. Thirdly, many studies did not report the socioeconomic profile of the included patients which may have a significant effect of the association between RB and HPV. Fourthly, the selection of the control samples (healthy controls are preferred) is of extreme importance for measuring the true association between HPV and RB. Fifthly, all studies were of fair criterion, therefore caution should be considered when interpreting our results.
