# Supplementary material for: Human Papillomavirus and Retinoblastoma: Evidence From a Systematic Review and Meta-analysis of Cross-Sectional Studies
Source: Int J Public Health. 2023 Jul 11;68:1605284. doi: 10.3389/ijph.2023.1605284 (PMC10366381; doi:10.3389/ijph.2023.1605284)
Supplement: Supplementary file 1 [file DataSheet1.docx]

| Table S1: Quality rating of the prospective cohort studies using National Institute of Health tool | | | | | | | | | | | | | | | |
| --- | --- | --- | --- | --- | --- | --- | --- | --- | --- | --- | --- | --- | --- | --- | --- |
| Reference ID | Q1 | Q2 | Q3 | Q4 | Q5 | Q6 | Q7 | Q8 | Q9 | Q10 | Q11 | Q12 | Q13 | Q14 | Total score |
| Anand-2015-India | 1 | 1 | 1 | 1 | 0 | 1 | 0 | 1 | 1 | 0 | 1 | 0 | 0 | 0 | 8 |
| Antoneli-2011-Brazil | 1 | 1 | 1 | 1 | 0 | 1 | 0 | 1 | 1 | 0 | 1 | 0 | 0 | 0 | 8 |
| Chauhan-2019-India | 1 | 1 | 1 | 1 | 0 | 1 | 0 | 1 | 1 | 0 | 1 | 0 | 0 | 0 | 8 |
| Jeyaprakash-2021-India | 1 | 1 | 1 | 1 | 0 | 1 | 0 | 1 | 1 | 0 | 1 | 0 | 0 | 0 | 8 |
| Mohan-2009-India | 1 | 1 | 1 | 1 | 0 | 1 | 0 | 1 | 1 | 0 | 1 | 0 | 0 | 0 | 8 |
| Fuentes-2003-Mexico | 1 | 1 | 1 | 1 | 0 | 1 | 0 | 1 | 1 | 0 | 1 | 0 | 0 | 0 | 8 |
| Naru-2016-India | 1 | 1 | 1 | 1 | 0 | 1 | 0 | 1 | 1 | 0 | 1 | 0 | 0 | 0 | 8 |
| Orjuela-2000-Mexico | 1 | 1 | 1 | 1 | 0 | 1 | 0 | 1 | 1 | 0 | 1 | 0 | 0 | 1 | 9 |
| Palazzi-2003-Brazil | 1 | 1 | 1 | 1 | 0 | 1 | 0 | 1 | 1 | 0 | 1 | 0 | 0 | 0 | 8 |
| Shetty-2012-India | 1 | 1 | 1 | 1 | 0 | 1 | 0 | 1 | 1 | 0 | 1 | 0 | 0 | 0 | 8 |
| Ryoo-2013-Korea | 1 | 1 | 1 | 1 | 0 | 1 | 0 | 1 | 1 | 0 | 1 | 0 | 0 | 0 | 8 |
| Saktanasate-Thailand-2018 | 1 | 1 | 1 | 1 | 0 | 1 | 0 | 1 | 1 | 0 | 1 | 0 | 0 | 1 | 9 |
| Javanmard-2019-Iran | 1 | 1 | 1 | 1 | 0 | 1 | 0 | 1 | 1 | 0 | 1 | 0 | 0 | 0 | 8 |
| Gillison-2007-Multicenter in North America | 1 | 1 | 1 | 1 | 0 | 1 | 0 | 1 | 1 | 0 | 1 | 0 | 0 | 0 | 8 |
| 1 yes, 0 = no, not applicable or not reported | | | | | | | | | | | | | | | |

**Journal:** International Journal of Public Health

**Title:** Human papillomavirus and retinoblastoma: evidence from a systematic review and meta-analysis of cross-sectional studies

Good (10-14 points), fair (6-9 points) and poor (0-5 points)

1. Was the research question or objective in this paper clearly stated?
2. Was the study population clearly specified and defined?
3. Was the participation rate of eligible persons at least 50%?
4. Were all the subjects selected or recruited from the same or similar populations (including the same time period)? Were inclusion and exclusion criteria for being in the study prespecified and applied uniformly to all participants?
5. Was a sample size justification, power description, or variance and effect estimates provided?
6. For the analyses in this paper, were the exposure(s) of interest measured prior to the outcome(s) being measured?
7. Was the timeframe sufficient so that one could reasonably expect to see an association between exposure and outcome if it existed?
8. For exposures that can vary in amount or level, did the study examine different levels of the exposure as related to the outcome (e.g., categories of exposure, or exposure measured as continuous variable)?
9. Were the exposure measures (independent variables) clearly defined, valid, reliable, and implemented consistently across all study participants?
10. Was the exposure(s) assessed more than once over time?
11. Were the outcome measures (dependent variables) clearly defined, valid, reliable, and implemented consistently across all study participants?
12. Were the outcome assessors blinded to the exposure status of participants?
13. Was loss to follow-up after baseline 20% or less?
14. Were key potential confounding variables measured and adjusted statistically for their impact on the relationship between exposure(s) and outcome(s)?

**Figure S1: Subgroup analysis of the prevalence of HPV in RB represented with the event rate and the corresponding 95% CI**

**
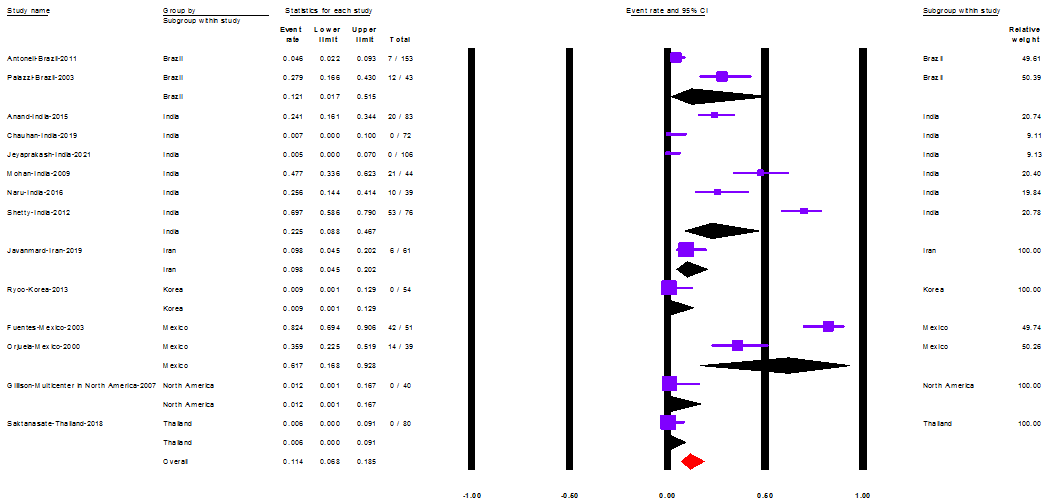
**

**Figure S2: The prevalence of HPV 16 in RB represented with the event rate and the corresponding 95% CI**

**
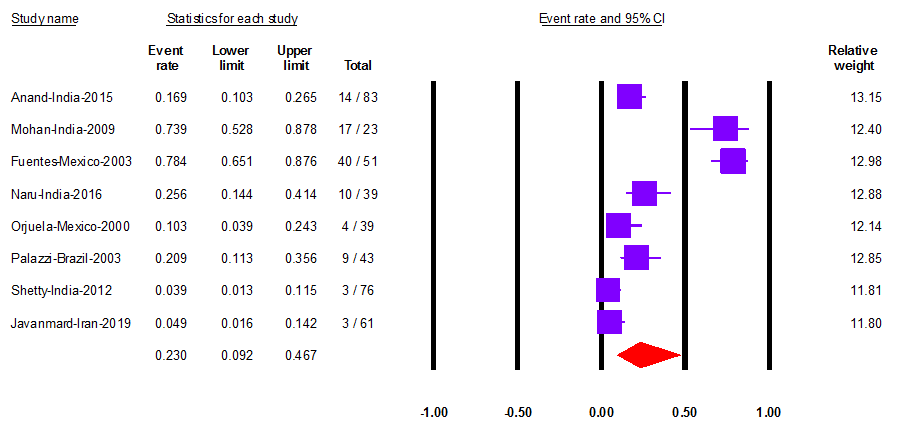
**

**Figure S3: The prevalence of HPV 18 in RB represented with the event rate and the corresponding 95% CI**

**
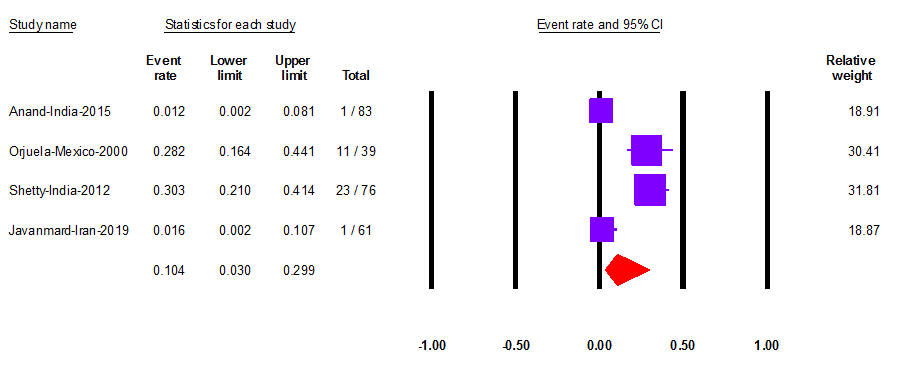
**

**Figure S4: The prevalence of HPV 16, 18 in RB represented with the event rate and the corresponding 95% CI**

**
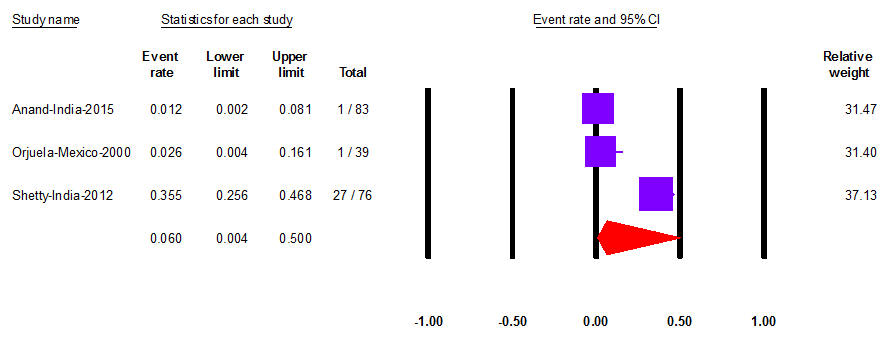
**

**Figure S5: The prevalence of HPV 35 in RB represented with the event rate and the corresponding 95% CI**

**
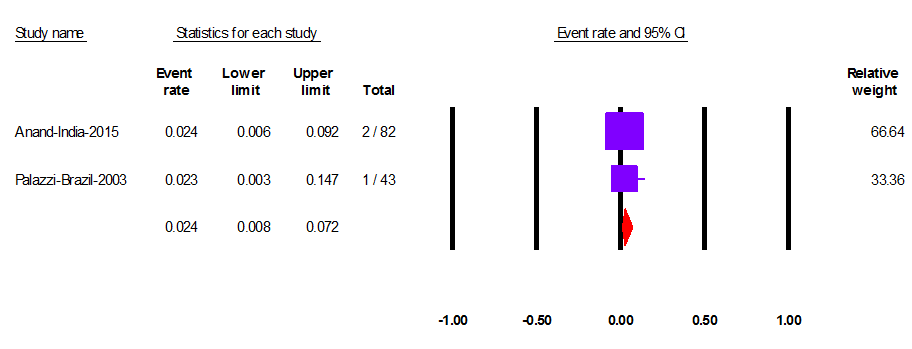
**

**Figure S6: The prevalence of HPV 11 in RB represented with the event rate and the corresponding 95% CI**

**
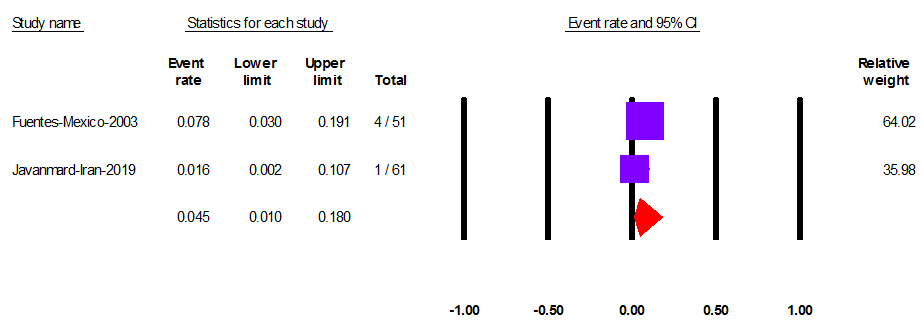
**
